# Differential immunomodulatory effects by Tripterygium wilfordii Hook f-derived refined extract PG27 and its purified component PG490 (triptolide) in human peripheral blood T cells: potential therapeutics for arthritis and possible mechanisms explaining in part Chinese herbal theory “Junn-Chenn-Zuou-SS”

**DOI:** 10.1186/1479-5876-11-294

**Published:** 2013-11-21

**Authors:** Ling-Jun Ho, Wen-Liang Chang, Ann Chen, Ping Chao, Jenn-Haung Lai

**Affiliations:** 1 Institute of Cellular and System Medicine, National Health Research Institute, Zhunan, Taiwan, R. O. C; 2 Graduate Institute of Basic Medical Science, PhD Program of Aging, China Medical University, Taichung, Taiwan, R. O. C; 3 School of Pharmacy, National Defense Medical Center, Taipei, Taiwan, R. O. C; 4 Department of Pathology, National Defense Medical Center, Taipei, Taiwan, R.O.C; 5 Institute of Molecular Biology, Academia Sinica, Taipei, R. O. C; 6 Division of Allergy, Immunology and Rheumatology, Department of Internal Medicine, Chang Gung Memorial Hospital, Chang Gung University, Tao-Yuan, Taiwan, R. O. C

**Keywords:** *Tripterygium wilfordii* Hook f, Nuclear factor kappaB, I-kappaBalpha kinase-beta, PG27, PG490 (Triptolide), T Cells

## Abstract

**Background:**

For thousands of years, it remains unclear why Chinese prefer complex herbal remedy and seldom try to purify it. One of the reasons is that they believe Chinese herbs compared to Western drugs are relatively less toxic and better tolerated. The so called “Junn-Chenn-Zuou-SS” theory illustrates a concept of coordinated effects from a combination of different Chinese herbs. PG27, a refined extract from a well-known Chinese antirheumatic herb *Tripterygium wilfordii* Hook f (TwHf), is effective in attenuating transplantation rejection and extending survival of cardiac xenografts.

**Methods:**

Experiments were conducted in human primary T lymphocytes isolated from buffy coat. The activities of the inhibitor of kappaB alpha kinase-inhibitor of kappaB alpha-nuclear factor kappaB (IKK-IκBα-NF-κB) and mitogen activated protein kinase-activator protein-1 (MAPK-AP-1) signaling pathways were determined via electrophoretic mobility shift assays, immunoprecipitation kinase assays, Western blots, and transfection assays.

**Results:**

We showed that PG27 inhibited IKKα-IκBα-NF-κB and MAPK-AP-1 signaling pathways; however, IKKβ activity was less susceptible to inhibition by PG27. In contrast, the purified component of TwHf, PG490 (triptolide), reduced both MAPK-AP-1 and IKK-IκBα-NF-κB signaling pathways, including both IKKα and IKKβ, with similar potency. By means of high performance liquid chromatography analysis, it was estimated that PG490 constituted 1.27 ± 0.06% of the total PG27 content. Further analysis demonstrated that compared to PG490 alone, PG27 that contained an equal amount of PG490 was less toxic and less immunosuppressive, suggesting the presence of cytoprotective ingredient(s) in the non-PG490 components of PG27.

**Conclusions:**

In addition to demonstrating the immunomodulatory capacity of PG27 as the potential therapeutics for arthritis and prevention of transplantation rejection, the differential regulatory effects and mechanisms by PG27 and PG490 further support in part a possibly-existing Chinese herbal theory “Junn-Chenn-Zuou-SS”.

## Background

According to the concept of Chinese herbal therapy, the greatest therapeutic effects come from a combination of several ingredients; some of them are effective in treating diseases and some of them modulate the function of these active components through enhancing their efficacy, reducing their side effects, or manipulating their delivery into the target organs. The record about this concept called “Junn-Chenn-Zuou-SS” is first published in *Shen Nong Ben Cao Jing*, an earliest medical material dictionary composed at the era of Qin and Han dynasties. The theory “Junn-Chenn-Zuou-SS” has been generally adopted in Chinese medicine practice where “Junn” represents the active effective ingredient(s); “Chenn” is the adjunctive ingredient(s) enhancing the effectiveness of active ingredient(s); “Zuou” is the complementary ingredient(s) reducing the side effects of active effective ingredient(s) and “SS” is the ingredient(s) helpful in delivering the active effective ingredient(s) into the target organs. The “Junn-Chenn-Zuou-SS” theory is presumably achieved by administering different herbs (called “Danfang” for each herb) with known functions together called a “Fufang” at a time. The concept of “Fufang” in fact has already been applied in medicine for a long time including the medications from Western societies. Typical examples include the treatments for rheumatoid arthritis (RA) [1] and HIV infection [2]. In these examples, a combination of several drugs preserving different inhibitory effects and mechanisms on specific molecules or pathways may work together and achieve synergistic effects. By doing this, the dosages of individual drugs may be greatly reduced to attenuate the potential toxic effects from each drug and yet the effects are synergistically enhanced. Nevertheless, there has been no scientific evidence showing that “Junn-Chenn-Zuou-SS” therapeutic theory may exist and work by different components in a single herb.

The most commonly used Chinese medicine for autoimmune disorders, such as RA, is *Tripterygium wilfordii* Hook f (TwHf; known as Thunder God Vine), which has potent immunosuppressive effects [3,4]. Currently, different TwHf extracts are prescribed to treat autoimmune disorders in mainland China. Aside from extensive clinical trials conducted in oriental populations, the double blinded studies in RA patients of Western populations also confirm its effectiveness [5,6]. In our previous work, we demonstrated that TwHf is an effective immunomodulatory drug, which acts by inhibiting T-cell activation and inducing T-cell apoptosis [7,8]. Although the usefulness of each ingredient of TwHf extracts has not been studied in detail, the major therapeutic effects of TwHf have been suggested to be from some of the ingredients such as PG490 (triptolide), tripdiolide, triptonide and triptophenolide [9-11].

Because the commonly prescribed TwHf preparations are considered to have toxicities, this greatly reduces the usefulness of this drug for clinical purposes. In order to minimize drug toxicity yet reserve drug efficacy, further purification of TwHf leads to the refined extract called PG27 that shows promising effects in prevention of bone marrow transplantation rejections [12]. Importantly, a combination of both PG27 and cyclosporine results in strong synergistic effects in extending the survival of hamster-to-rat cardiac xenograft model [13]. In this context, the TwHf purified product PG490 also preserves strong immunosuppressive effects [14,15]. In the light of the current therapeutic strategy for autoimmune disorders with a combination of several disease-modifying antirheumatic drugs to increase efficacy and to reduce adverse events [1], the exploration of effects and mechanisms of Chinese antirheumatic drugs should bring more alternatives for the therapy of autoimmune disorders.

The nuclear factor kappaB (NF-κB) family consists of Rel-domain-containing proteins that are crucial for the regulation of inflammation and immune responses [16,17]. In resting cells, these proteins are retained in the cytosol by a group of inhibitory proteins such as inhibitor of kappaB alpha (IκBα). After activation, IκBα is phosphorylated by IκBα kinases (IKKs) such as IKKα and IKKβ, and undergoes ubiquitination and subsequent proteosome-mediated degradation [18]. This leads to the nuclear translocation of NF-κB from the cytosol and induces the activation of a variety of genes, leading to diseases such as RA [19]. Similar to NF-κB, the activating protein-1 (AP-1) transcription factors, which consist of Jun and Fos family proteins, extensively participate in regulating cell proliferation, transformation and death, and serve as good therapeutic targets for the control of inflammation [20].

In this report, we investigated the effects and mechanisms of PG27-mediated immunomodulation in primary T cells. We observed that PG27 had differential immunosuppressive potency for IKKα and IKKβ, a phenomenon not observed for the purified compound PG490. In addition, PG27 that contained an equivalent amount of PG490 was less toxic than PG490 alone. These observations explain in part a possible mechanism of “Junn-Chenn-Zuou-SS” theory and provide evidence suggesting that PG27 may be assessed for potential use as a disease-modifying antirheumatic drug for autoimmune disorders like RA.

## Materials and methods

### Preparation of PG27 and PG490

PG27 powder was prepared by Pharmagenesis (La Jolla, California) and was kindly provided by PhytoHealth, Inc, Taipei, Taiwan. PG490 was purchased from Sigma-Aldrich Chemical Company (St. Louis, MO). Both drugs were dissolved in DMSO to generate 100 mg/mL (PG27) or 100 ng/mL (PG490) stock solutions. For experiments, the required concentrations of each drug were made by dilution of the concentrated stock solution with culture medium, which contained RPMI 1640 medium supplemented with 10% fetal bovine serum, 2 mM glutamine, and 1,000 U/mL penicillin-streptomycin (Gibco-BRL, Gaithersberg, MD).

### Preparation of peripheral blood T cells

The use of buffy coat purchased from the blood bank (Taipei, Taiwan) was approved by the Institutional Review Board, Tri-Service General Hospital, through a fast review tract. Human peripheral blood T cells were purified from whole blood via negative selection, according to our previous report [8]. Briefly, buffy coat was mixed with Ficoll-Hypaque, and the layer of mononuclear cells was collected after centrifugation. After lysis of red blood cells, the peripheral blood mononuclear cells were incubated with antibodies (Abs), including L243 (anti-DR; American Type Culture Collection [ATCC], Rockville, MD), OKM1 (anti-CD11b; ATCC), and LM2 (anti-Mac1; ATCC) for 30 min at 4°C. The cells were washed with medium containing 10% fetal bovine serum and incubated with magnetic beads conjugated to goat anti-mouse IgG (R & D, Minneapolis, MN). The antibody-stained cells were then removed with a magnet. Following a repeat of the above procedures, T cells were obtained with a purity of more than 98%, as determined by the percentage of CD3^+^ cells in flow cytometry (Beckton Dickinson, Mountain View, CA).

### Cell stimulation, cytokine determination, and cell survival measurement

To activate T cells, the following stimuli were used: phorbol 12-myristate 13-acetate (PMA; Sigma) at 10 ng/mL, ionomycin (Sigma) at 1 μM, immobilized monoclonal antibody (mAb) anti-CD3 (OKT3; ATCC) at 10 μg/mL, soluble anti-CD28 mAb (Beckton Dickinson) at 1 μg/mL, and TNF-α at 10 ng/mL. The cells were incubated with the various stimuli, and the cell pellets or supernatants were collected for analysis. The determination of cytokine concentrations was performed via ELISA as described previously [21]. IC_50_ was the concentration of drug (PG27 or PG490) that inhibited half the cytokine production from different stimuli-activated T cells and was calculated by linear regression using Microsoft Excel. Cell viability was measured by either the trypan blue exclusion assay or the MTT (3-[4,5-Dimethylthiazol-2-yl]-2,5-diphenyl-tetrazolium bromide) colorimetric assay, as described in our previous report [8]. Similarly, the 50% lethal concentrations (LC_50_) of PG27 and PG490 were calculated.

### Preparation of cytoplasmic and nuclear extracts

Cytoplasmic and nuclear extracts were prepared according to our published work [21]. Briefly, treated cells were incubated at 4°C in 50 μL of buffer A (10 mM HEPES, pH 7.9, 10 mM KCl, 1.5 mM MgCl_2_, 1 mM DTT, 1 mM PMSF, 3.3 μg/mL aprotinin) for 15 min, with occasional gentle vortexing. The swollen cells were centrifuged at 15,000 rpm for 3 min. After removal of the supernatants (cytoplasmic extracts), the pelleted nuclei were washed with 50 μL of buffer A, and subsequently, the cell pellets were resuspended in 30 μL of buffer C (20 mM HEPES, pH 7.9, 420 mM NaCl, 1.5 mM MgCl_2_, 0.2 mM EDTA, 25% glycerol, 1 mM DTT, 0.5 mM PMSF, 3.3 μg/mL aprotinin) and incubated at 4°C for 30 min, with occasional vigorous vortexing. The mixtures were then centrifuged at 15,000 rpm for 20 min, and the supernatants were used as nuclear extracts.

### Electrophoretic mobility shift assays (EMSAs)

EMSAs were performed as detailed in our previous report [21]. Oligonucleotides containing the NF-κB-binding site (5′-AGT TGA GGG GAC TTT CCC AGG C-3′), the AP-1-binding site (5′-CGC TTG ATG AGT CAG CCG GAA-3′), and the Oct-1-binding site (5′-TGT CGA ATG CAA ATC ACT AGA A-3′) were purchased and used as DNA probes (Promega, Madison, WI). The DNA probes were radio-labeled with [γ-^32^P]ATP using T4 kinase, according to the manufacturer’s instructions (Promega). For the binding reactions, the radio-labeled probes were incubated with 5 μg of nuclear extracts. The binding buffer contained 10 mM Tris–HCl, pH 7.5, 50 mM NaCl, 0.5 mM EDTA, 1 mM DTT, 1 mM MgCl_2_, 4% glycerol, and 2 μg poly(dI-dC). The reaction mixtures were incubated at room temperature for 20 min prior to the binding reaction. For supershift assays, different mAbs were preincubated with nuclear extracts for 30 min before the addition of the radiolabeled probes. The final reaction mixtures were analyzed in 6% non-denaturing polyacrylamide gels with 0.25× Tris-borate/EDTA as an electrophoresis buffer.

### Western blotting

ECL Western blotting (Amersham, Arlington Heights, IL) was performed as described previously [8]. Briefly, equal amounts of whole cell lysates and cytoplasmic or nuclear extracts were analyzed by 10% SDS-PAGE and transferred to a nitrocellulose filter. For immunoblotting, the nitrocellulose filter was incubated with Tris-buffered saline containing 5% nonfat milk (milk buffer) for 2 h, and then blotted with antisera against IκBα, IKKα, IKKβ (Santa Cruz Biotechnology), or β-actin overnight at 4°C. After washing twice with milk buffer, the filter was incubated with donkey anti-mouse IgG conjugated to horseradish peroxidase at a concentration of 1:5,000 for 30 min. The filter was then incubated with the substrate and exposed to X-ray film.

### Immunoprecipitation kinase assay

The immunoprecipitation kinase assay was described in detail in our previous report [21]. The GST-IκBα fusion protein was used as a substrate for IKKα and IKKβ. The JNK substrate, a GST-c-Jun fusion protein, was a kind gift from Dr. S.-F. Yang (Academia Sinica, Taiwan). Myelin basic protein (MBP), which was used as a substrate for both ERK and p38, was purchased from Sigma. The Abs used for the kinase assays were purchased from Cell Signaling (Beverly, MA; for anti-JNK and anti-p38 polyclonal Abs) and Santa Cruz Biotechnology (for anti-ERK, anti-IKKα and anti-IKKβ polyclonal Abs). To perform the immunoprecipitation kinase assay, 50–100 μg of whole cell extract was incubated with 5 μL of specific Abs in an incubation buffer (25 mM HEPES, pH 7.7, 300 mM NaCl, 1.5 mM MgCl_2_, 0.2 mM EDTA, 0.1% Triton-X-100, 20 mM β-glycerophosphate, 0.1 mM Na_3_VO_4_, 2 μM leupeptin, 400 μM PMSF) overnight. The mixture was then immunoprecipitated by the addition of protein A beads and rotated at 4°C for 2 h. After extensive washing, (twice with a HEPES washing buffer [20 mM HEPES, pH 7.7, 50 mM NaCl, 2.5 mM MgCl_2_, 0.1 mM EDTA, 0.05% Triton X-100]; twice with an LiCl washing buffer [500 mM LiCl, 100 mM Tris, pH 7.6, 0.1% Triton X-100, 1 mM DTT]; and twice with a kinase buffer [20 mM MOPS, pH 7.2, 2 mM EDTA, 10 mM MgCl_2_, 0.1% Triton X-100 and 1 mM DTT], the beads were resuspended in 40 μL of kinase buffer, along with cold ATP (30 μM) and 10 μCi of [γ-^32^P]ATP. The mixture was incubated at 30°C with occasional gentle mixing for 30 min. The reaction was then terminated by resuspending the samples in a 1% SDS solubilizing buffer and boiling for 5 min. The samples were then analyzed by SDS-PAGE.

### Transfection assay in purified human peripheral blood T cells

The transfection assay was performed by electroporation with an Amaxa Nucleofector apparatus, according to the manufacturer’s instructions (Amaxa, Cologne, Germany). In brief, primary T cells were mixed with 5 μg of the reporter plasmid pNF-κB-luciferase (Luc) or pAP-1-Luc (Stratagene, La Jolla, CA) in 100 μL of the provided electroporation buffer. After electroporation, the cells were transferred to 2 mL of pre-warmed RPMI medium. After transfection for 48 h, the cells were aliquoted equally for testing the individual conditions described in the figure legends. For treatment, the drugs were added 2 h before the addition of stimuli. After stimulation with TNF-α for 18 h, the cell pellets were collected, total cell lysates were prepared, and the luciferase activity, after normalization to the total protein amounts, was determined according to the manufacturer’s instructions (Promega).

### Analysis of the PG490 content in PG27 by HPLC

Both PG490 (1.6 mg) and PG27 (2.4 mg) were dissolved in DMSO (1.0 mL) to generate stock solutions. PG490 was spiked into the mobile phase to create a series of standards consisting of 0.0125, 0.025, 0.05, 0.1, 0.2, 0.4, 0.8, and 1.6 mg/mL concentrations. High Performance Liquid Chromatography (HPLC) was performed on an Agilent Model 1100 Quat pump system equipped with an Agilent 1100 VWD Detector and an Agilent 1100 ALS auto-injector. The detector was set to 218 nm. A reversed phase column (Cosmosil 5C18-AR-II, 25 cm × 4.6 mm I.D.) was applied. The mobile phase was 45% MeOH/55% H_2_O eluted at a flow rate of 0.8 mL/min. The injection volume was 20 μL for each sample.

### Statistics

The results were expressed as means ± standard deviations. One-way ANOVA analysis was used to evaluate the differences, which were considered to be statistically significant at a P value of <0.05.

## Results

### PG27 inhibited IL-2 production from activated T cells

**Figure 1:**
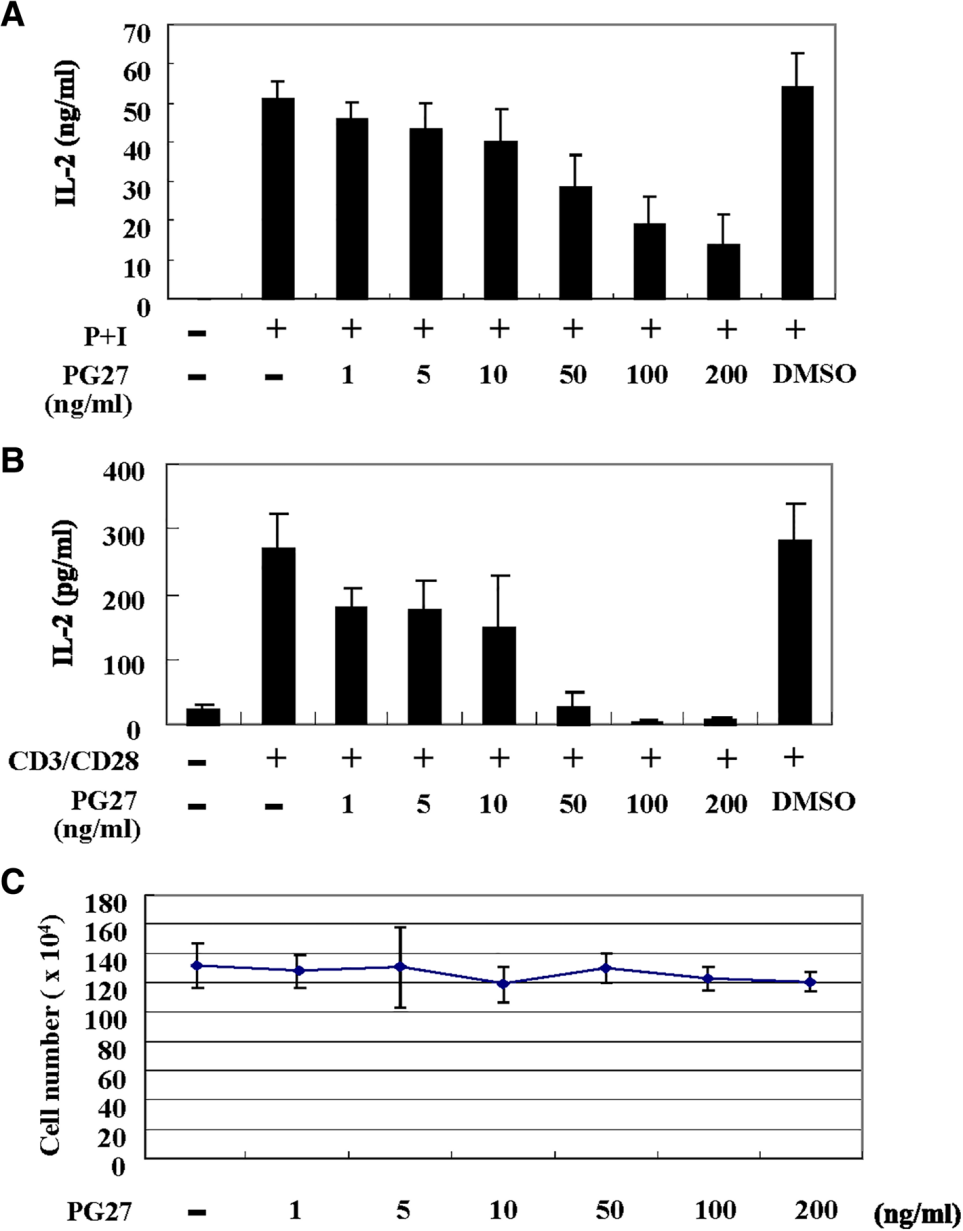
**PG27 inhibited IL-2 production from activated T cells in a concentration-dependent manner.** Human peripheral blood T cells at a concentration of 1 × 10^6^/mL were pretreated with various concentrations of PG27 for 2 h and then stimulated with PMA + ionomycin (shown as P + I) **(A)** or CD3/CD28 **(B)** for 24 h. The supernatants were collected for the determination of IL-2 concentrations. In parallel, cell survival in the various conditions was determined by trypan blue exclusion assays **(C)**. The results shown are from independent experiments. Each experiment was performed in triplicate using T cells from at least 3 different donors.

To determine whether PG27 exhibited immunosuppressive effects that were as potent as the crude TwHf extract, purified T cells were activated with various stimuli in the presence or absence of PG27, and IL-2 concentrations were determined by ELISA (Figure 1). PG27 inhibited IL-2 production induced by PMA + ionomycin (Figure 1A) or CD3/CD28 (Figure 1B) in a concentration-dependent manner. There was no detectable cytotoxicity, in the presence or absence of stimulus, resulting from the tested concentrations of PG27 (Figure 1C and data not shown).

### PG27 inhibited NF-κB and AP-1 activation induced by various stimuli

**Figure 2:**
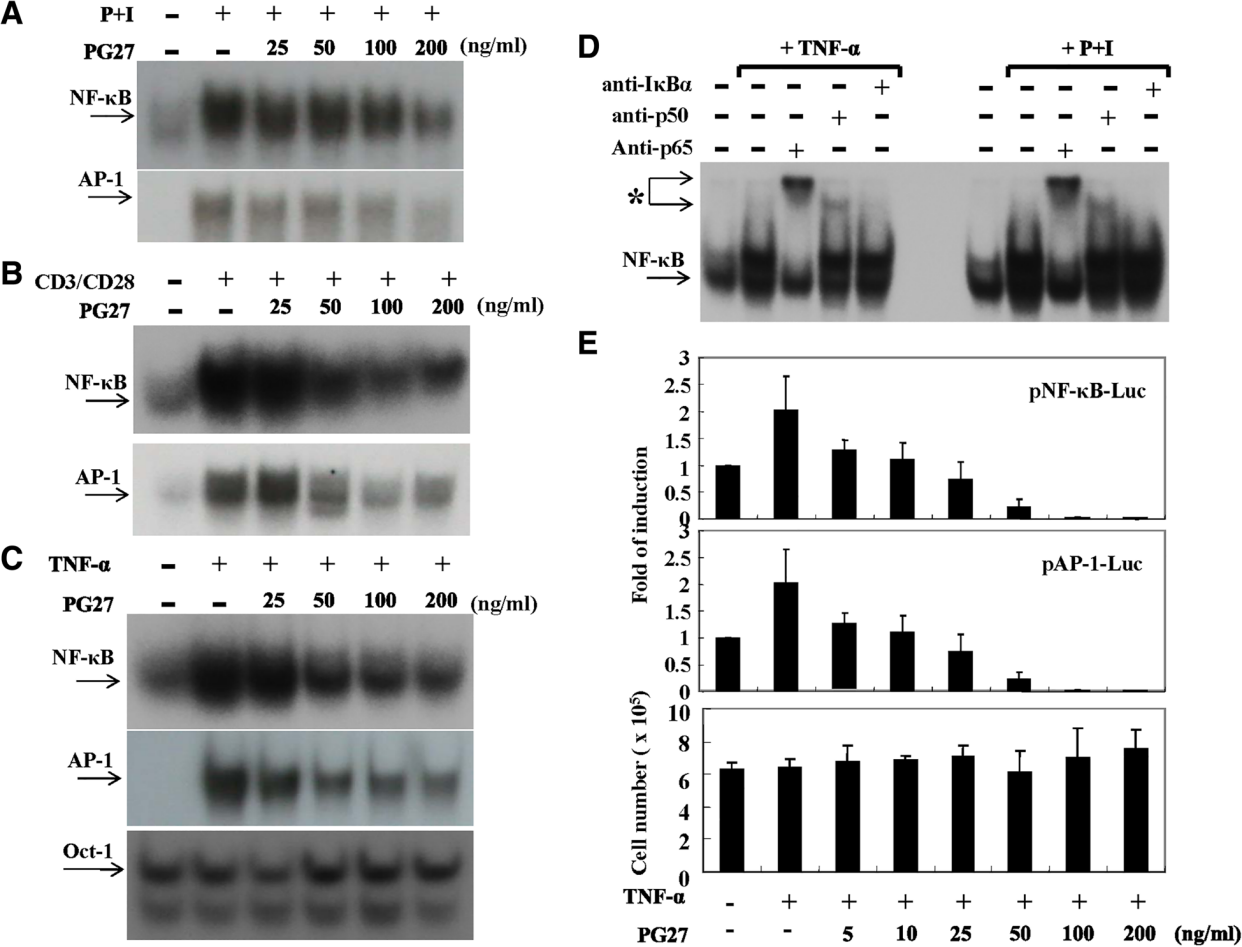
**PG27 inhibited NF-κB and AP-1 activation induced by various stimuli.** Human peripheral blood T cells at a concentration of 2 × 10^6^/mL were pretreated with various concentrations of PG27 for 2 h and then stimulated with PMA + ionomycin for 2 h **(A)**, CD3/CD28 for 6 h **(B)** or TNF-α for 6 h **(C)**. The nuclear extracts were prepared and analyzed for both NF-κB and AP-1 DNA-binding activity by EMSA. As a control, the DNA-binding activity of Oct-1 was measured **(C)**. In **(D)**, before adding the radiolabeled oligonucleotides to the reaction mixture, the nuclear extracts were preincubated with 5 μl of the indicated mAb for 30 min. The asterisk indicates the supershifted bands. In **(E)**, T cells at a concentration of 1 × 10^6^/mL were mixed with pNF-κB-Luc or pAP-1-Luc reporter plasmids and transfection reagents. The transfection was performed using an Amaxa Nucleofector according to the manufacturer’s instructions. After transfection for 48 h, the cells were aliquoted equally for the individual conditions and pretreated with various concentrations of PG27 for 2 h. After stimulation with TNF-α for another 18 h, cells were collected and total cell lysates were analyzed for luciferase activity. Cell survival was determined by trypan blue exclusion assays. Representative data of at least 3 independent experiments are shown.

We next examined the potential effects of PG27 on the activation of NF-κB and AP-1. After the treatment with different stimuli in the presence or absence of various concentrations of PG27, T cells were collected and nuclear extracts were prepared and analyzed by EMSA. As shown in Figure 2, PG27 suppressed PMA + ionomycin-induced (Figure 2A) and CD3/CD28-induced (Figure 2B) NF-κB and AP-1 DNA-binding activities. Considering the important role of TNF-α in inflammatory response, we also examined the effects of PG27 on TNF-α-stimulated T cells. Consistent with the experimental results for the previous stimuli, PG27 efficiently reduced the TNF-α-induced NF-κB and AP-1 DNA-binding activities (Figure 2C). In contrast, PG27 did not affect the DNA-binding activity of the negative control Oct-1 (Figure 2C). Furthermore, the highest concentration of the solvent DMSO used to dissolve PG27 did not show any inhibitory effect on NF-κB or AP-1 DNA-binding activity (data not shown). Using EMSA supershift analysis, performed in the presence of anti-p65 or anti-p50 mAbs, we further demonstrated that the transcription factors induced by TNF-α and PMA + ionomycin that bound the radiolabeled κB oligonucleotide probes consisted of at least p65 and p50 (Figure 2D). These results are consistent with the observations demonstrated in our previous report examining nuclear extracts from CD3/CD28-stimulated T cells [21]. To assess whether PG27 inhibited the transcription of genes containing binding sites for NF-κB and AP-1, we transiently transfected NF-κB- or AP-1-luciferase reporter constructs into purified human peripheral blood T cells. Forty-eight hours after transfection, T cells were aliquoted equally for the treatment with various concentrations of PG27 and then stimulated with TNF-α as indicated (Figure 2E). The total cell lysates were prepared and the luciferase activities were determined. The data showed that PG27 effectively suppressed the transcriptional activities of both NF-κB and AP-1 induced by TNF-α (Figure 2E). Side-by-side examinations of cell viability revealed no significant cytotoxicity of PG27 in all conditions tested (Figure 2E).

### Effects of PG27 on IκBα degradation and IKK activity

**Figure 3:**
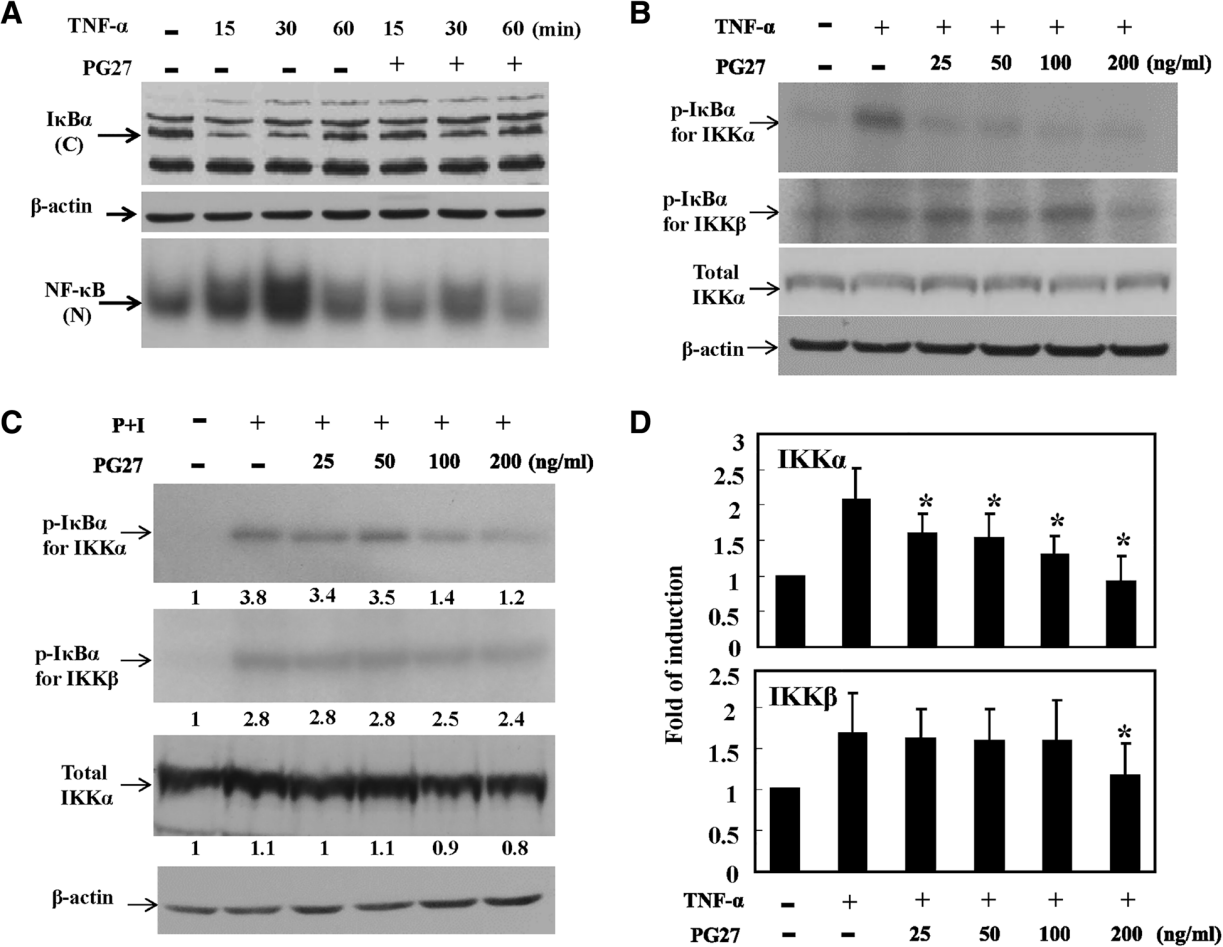
**PG27 prevented IκBα degradation and reduced the activity of IKKα but not IKKβ.** In **(A)**, T cells were pretreated with PG27 (200 ng/mL) for 2 h or left untreated, and then stimulated with TNF-α for various lengths of time as indicated. After washing, cell pellets were collected and cytoplasmic and nuclear extracts were prepared and analyzed for the protein levels of IκBα by Western blotting (upper panel) and NF-κB DNA-binding activity by EMSA (lower panel), respectively. In **(B)**, T cells were pretreated with PG27 for 2 h and then stimulated with TNF-α for 10 min. After washing, cell pellets were collected and total cell lysates were prepared and analyzed for the kinase activity of IKKα (upper panel) or IKKβ (middle panel) by immunoprecipitation kinase assays. The total cell lysates were also analyzed for IKKα protein levels by Western blotting (lower panel). In **(C)**, after pretreatment with PG27, T cells were stimulated with PMA + ionomycin for 15 min, and the cell lysates were collected for measurement of IKKα and IKKβ activity. The densitometric intensities of the individual bands are shown. The analysis of PG27-mediated suppression of TNF-α-stimulated IKKα and IKKβ was performed in T cells from at least 3 different donors **(D)**. *, P value of <0.05.

The upstream signaling molecules that control the activation of NF-κB were examined. T cells were treated with TNF-α for 15, 30, or 60 min in the presence or absence of PG27, and then cytoplasmic extracts were prepared to determine IκBα levels by Western blotting. As shown in Figure 3A, the cytosolic levels of IκBα decreased after TNF-α stimulation for 15 min; however, the pre-treatment with PG27 maintained IκBα levels close to the basal level. When nuclear extracts were examined for NF-κB DNA-binding activity, similar levels of suppression by PG27 were observed (Figure 3A). Considering that the regulation of IκBα protein levels depends on the activity of IKKs, which phosphorylate IκBα leading to its ubiquitination and degradation, we next wanted to determine whether PG27 could suppress IKK activity. To this end, immunoprecipitation kinase assays were performed with GST-IκBα as a substrate for both IKKα and IKKβ. As shown in Figure 3B, PG27 at a wide range of concentrations suppressed TNF-α-induced IKKα activity. In contrast, TNF-α-induced IKKβ activity was unexpectedly resistant to PG27 treatment; suppression was observed only mildly under the highest concentration of PG27 (Figure 3B). The differential suppression of IKKα and IKKβ activity by PG27 was not due to unequal loading of total protein, because the basal IKKα and β–actin levels appeared to be very similar for each sample (Figure 3B). To determine whether the differential suppression of IKKα and IKKβ by PG27 was specifically limited to TNF-α-induced signaling, additional immunoprecipitation kinase assays were performed using the PMA + ionomycin stimulus to treat cells. In agreement with the results for TNF-α stimulation, PG27 also showed preferential suppression of PMA + ionomycin-induced IKKα activity (Figure 3C). The PG27-mediated differential suppression of TNF-α-induced IKKα and IKKβ activities was statistically analyzed and the results suggested that PG27, at concentrations from 25 to 200 ng/mL, effectively inhibited TNF-α-induced IKKα activity; however, the suppression of IKKβ activity was observed only mildly under the highest PG27 concentration (Figure 3D).

### PG490 inhibited NF-κB and AP-1 activation

**Figure 4:**
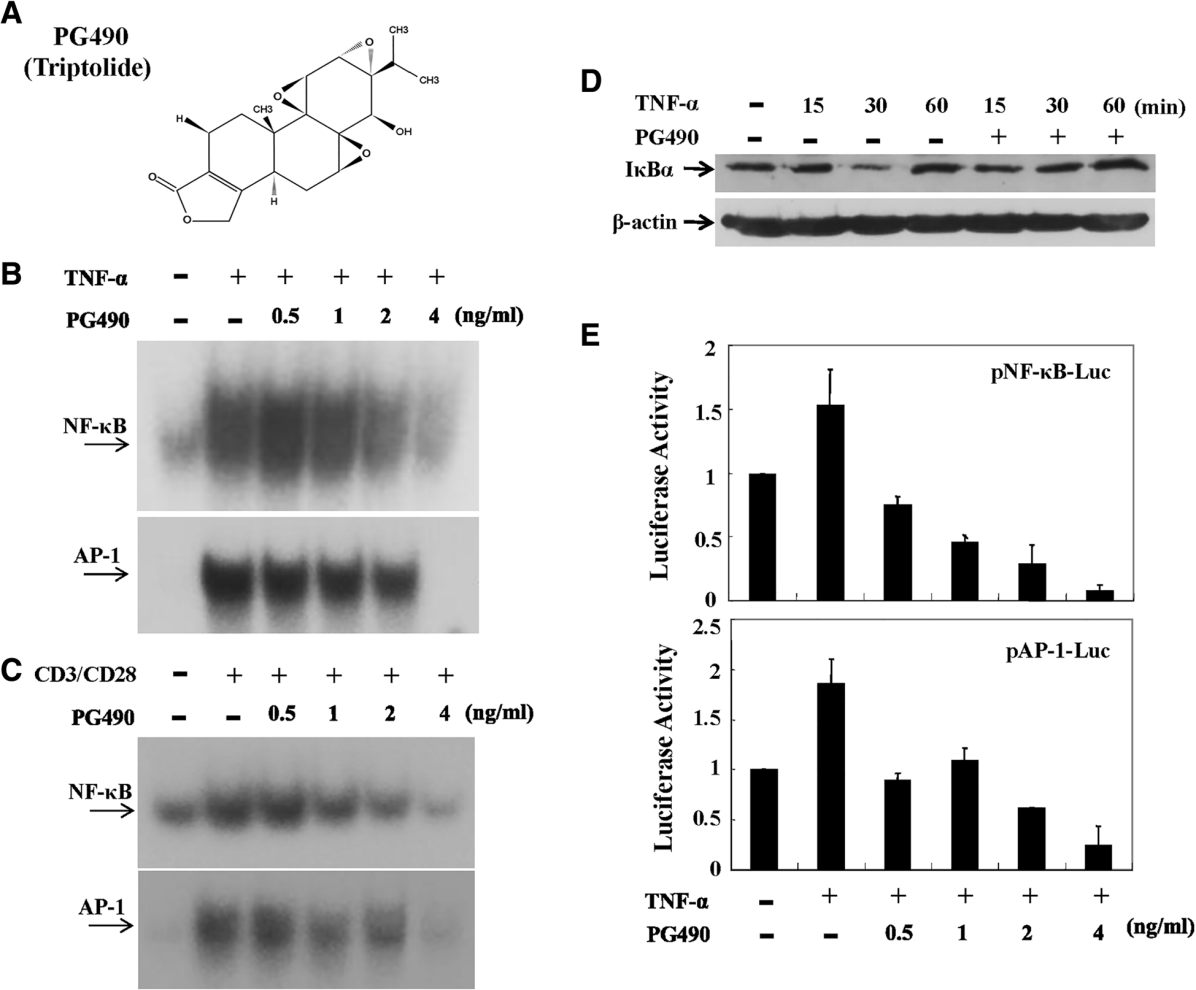
**PG490 inhibited both NF-κB and AP-1 DNA-binding and transcriptional activity.** T cells were pretreated with various concentrations of PG490 **(A)** for 2 h and then stimulated with TNF-α **(B)** or CD3/CD28 **(C)** for 6 h. The nuclear extracts were analyzed for both NF-κB and AP-1 DNA-binding activity by EMSA. In **(D)**, T cells were pretreated with PG490 for 2 h or left untreated, and then stimulated with TNF-α for various lengths of time as indicated. After washing, cell pellets were collected and cytoplasmic extracts were analyzed for the protein levels of IκBα and β-actin by Western blotting. In **(E)**, T cells were mixed together with pNF-κB-Luc or pAP-1-Luc reporter plasmids and the transfection procedures were performed as described for Figure 2. After electroporation for 48 h, the cells were aliquoted equally and pretreated with various concentrations of PG490 for 2 h. After stimulation with TNF-α for another 18 h, cells were collected and analyzed for luciferase activity. Representative data of at least 3 independent experiments are shown.

The unexpected differential effects of PG27 on IKK activity led us to investigate the effects of the purified TwHf component, PG490 (Figure 4A). T cells were treated with various concentrations of PG490 and then stimulated with TNF-α. Nuclear extracts were then prepared and the DNA-binding activities of NF-κB and AP-1 was analyzed by EMSA. As shown in Figure 4B, PG490 reduced the TNF-α-induced DNA-binding activities of both NF-κB and AP-1. Similar suppressive effects of PG490 were observed when the stimulus TNF-α was replaced by CD3/CD28 (Figure 4C). We also wanted to determine whether PG490 was able to regulate the expression of IκBα, which controls NF-κB activation. Western blots indicated that PG490 effectively inhibited the degradation of IκBα induced by TNF-α (Figure 4D). Consistent with these data, transfection assays demonstrated the suppressive potency of PG490 on the transcriptional activity of NF-κB and AP-1 (Figure 4E) induced by TNF-α.

### PG490 inhibited both IKKα and IKKβ activities

**Figure 5:**
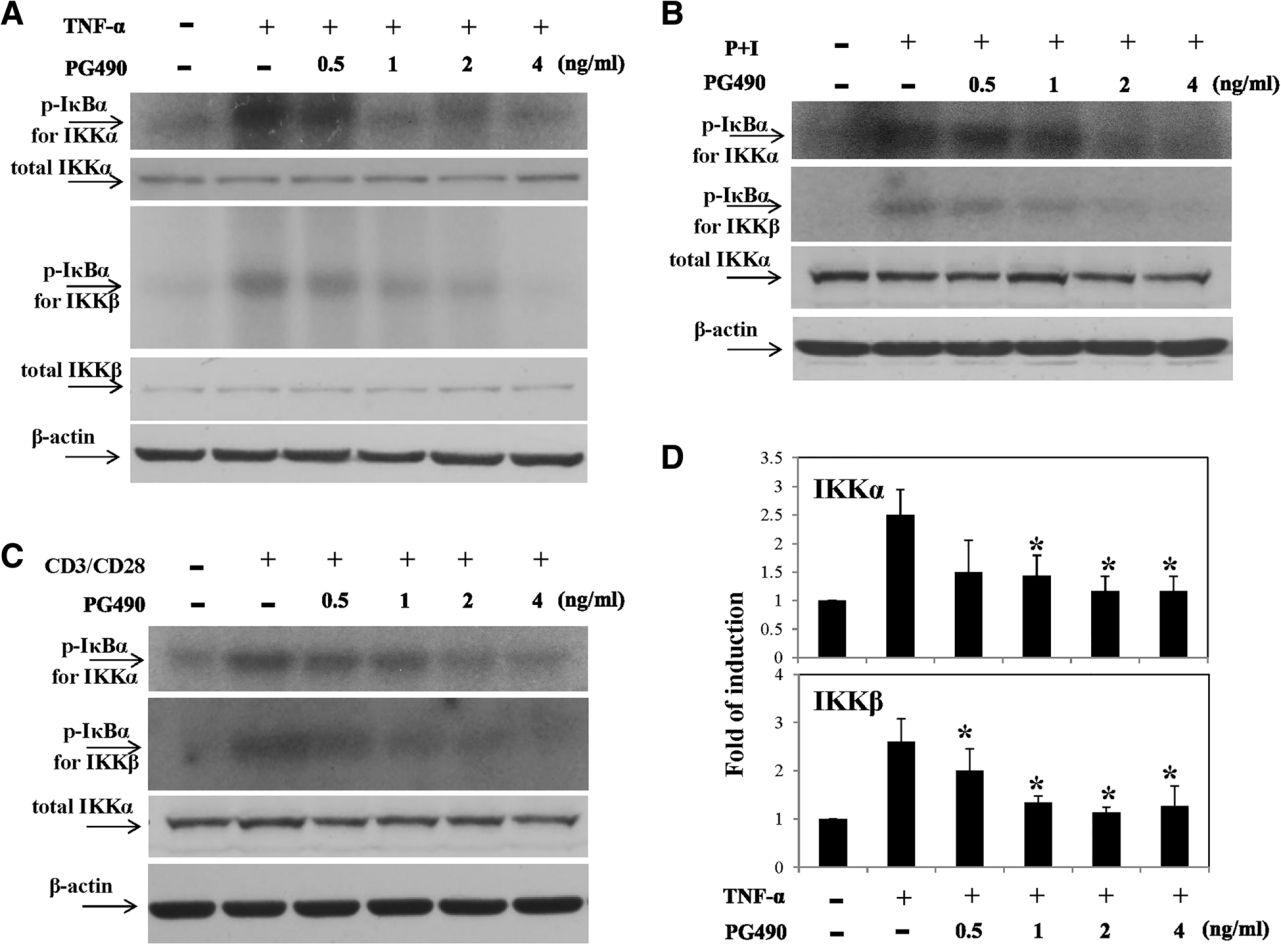
**PG490 inhibited both IKKα and IKKβ kinase activity.** T cells were pretreated with various concentrations of PG490 for 2 h and then stimulated with TNF-α for 10 min **(A)**, PMA + ionomycin for 15 min **(B)** or CD3/CD28 for 30 min **(C)**. Cell pellets were collected and total cell lysates were prepared and analyzed for the kinase activity of IKKα and IKKβ by immunoprecipitation kinase assays. The total IKKα and/or total IKKβ levels were determined by Western blotting. The analysis of PG490-mediated suppression of IKKα and IKKβ activities induced by TNF-α stimulation was performed on pooled data from T cells from 3 different donors **(D)**. *, P value of <0.05.

To determine whether PG490 was similar to PG27 in its inhibitory effects on TNF-α-induced IKKα and IKKβ, immunoprecipitation kinase assays were performed. Surprisingly, unlike PG27, PG490 reduced the kinase activities of both IKKα and IKKβ to a similar extent (Figure 5A). To investigate whether the effects of PG490 were observed only in T cells stimulated by TNF-α, different T cell stimuli were also examined. Consistent with the observations in TNF-α-stimulated cells, the kinase activities of both IKKα and IKKβ induced by PMA + ionomycin (Figure 5B) or CD3/CD28 (Figure 5C) was comparably inhibited by PG490 in a concentration-dependent manner. These results indicated that the suppression of both IKKα and IKKβ by PG490 was generally observed in human peripheral blood T cells activated by different stimuli. For consistency, the PG490-mediated suppression of IKKα and IKKβ activities induced by TNF-α stimulation in T cells from 3 different donor blood was quantitatively measured (Figure 5D).

### PG27 and PG490 downregulated MAPK activity

**Figure 6:**
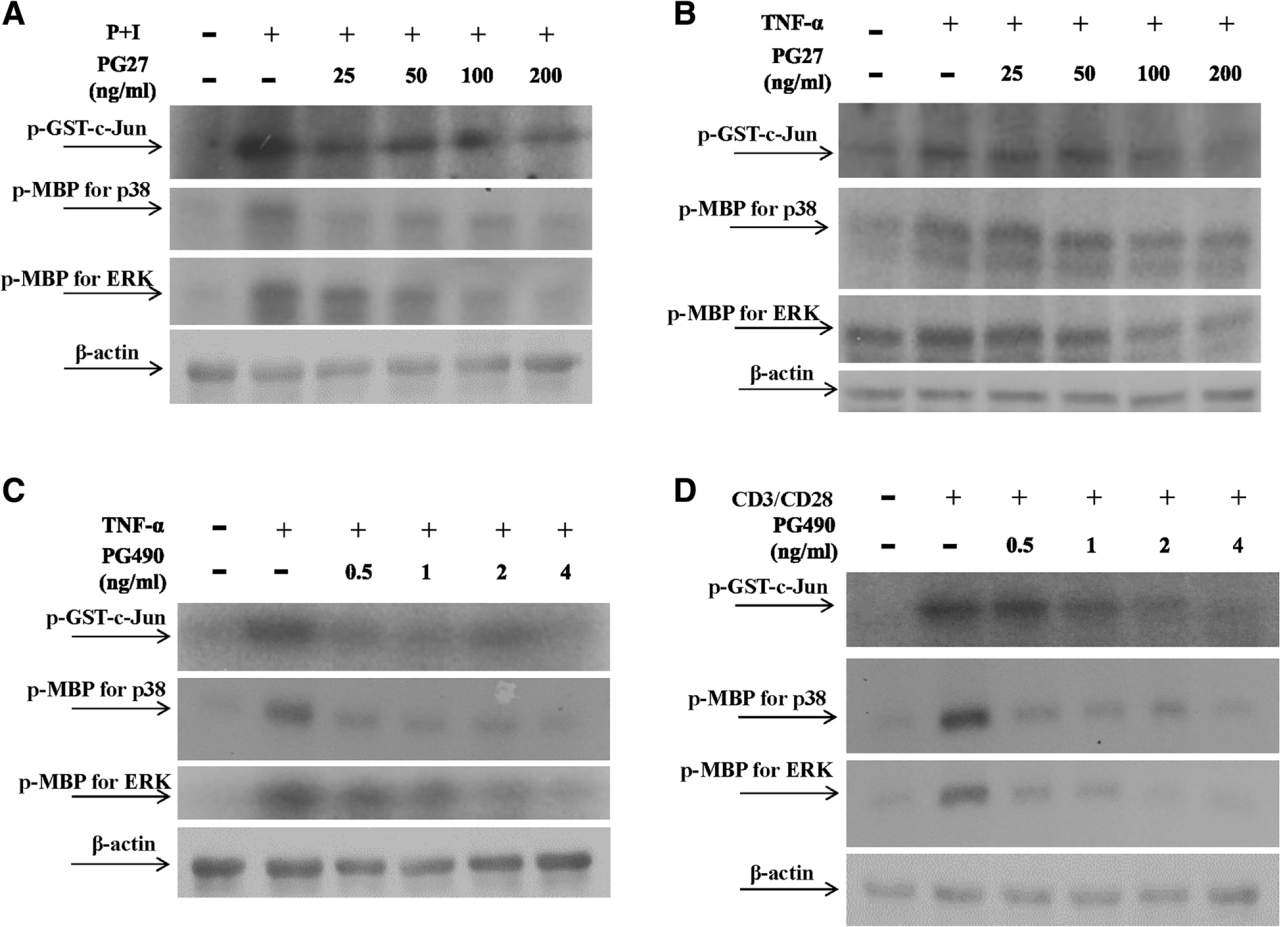
**PG27 and PG490 down regulated MAPK activity.** T cells at a concentration of 5 × 10^6^/mL were pretreated with various concentrations of PG27 **(A and B)** or PG490 **(C and D)** for 2 h and then stimulated with PMA + ionomycin for 30 min **(A)**, TNF-α for 10 min **(B and C)** or CD3/CD28 for 30 min **(D)**. Cell pellets were collected and total cell lysates were immunoprecipitated with anti-JNK, anti-p38 or anti-ERK antibodies. After sequential washes, the substrates (GST-c-Jun for JNK and MBP for both p38 and ERK) and [γ-^32^P]ATP were added individually. After the kinase reaction, the mixture was analyzed by SDS-PAGE. Representative data of at least 3 independent experiments are shown.

Although both PG27 and PG490 could successfully inhibit activation of NF-κB, they preserved differential suppressive activity against NF-κB upstream signaling molecules IKKα and IKKβ. Given that PG27 and PG490 could also effectively inhibit AP-1 activity, we determined whether they might have different suppressive potency on AP-1 upstream signaling kinases. Interestingly, as comparisons, the results showed that both PG27 and PG490 could effectively suppress MAP kinases, including JNK, p38 and ERK activities induced by various stimuli, albeit to different degrees (Figure 6A,B,C and D).

### Side-by-side comparisons of the effects of PG27 and PG490 on IL-2 production and cytotoxicity in activated T cells

**Figure 7:**
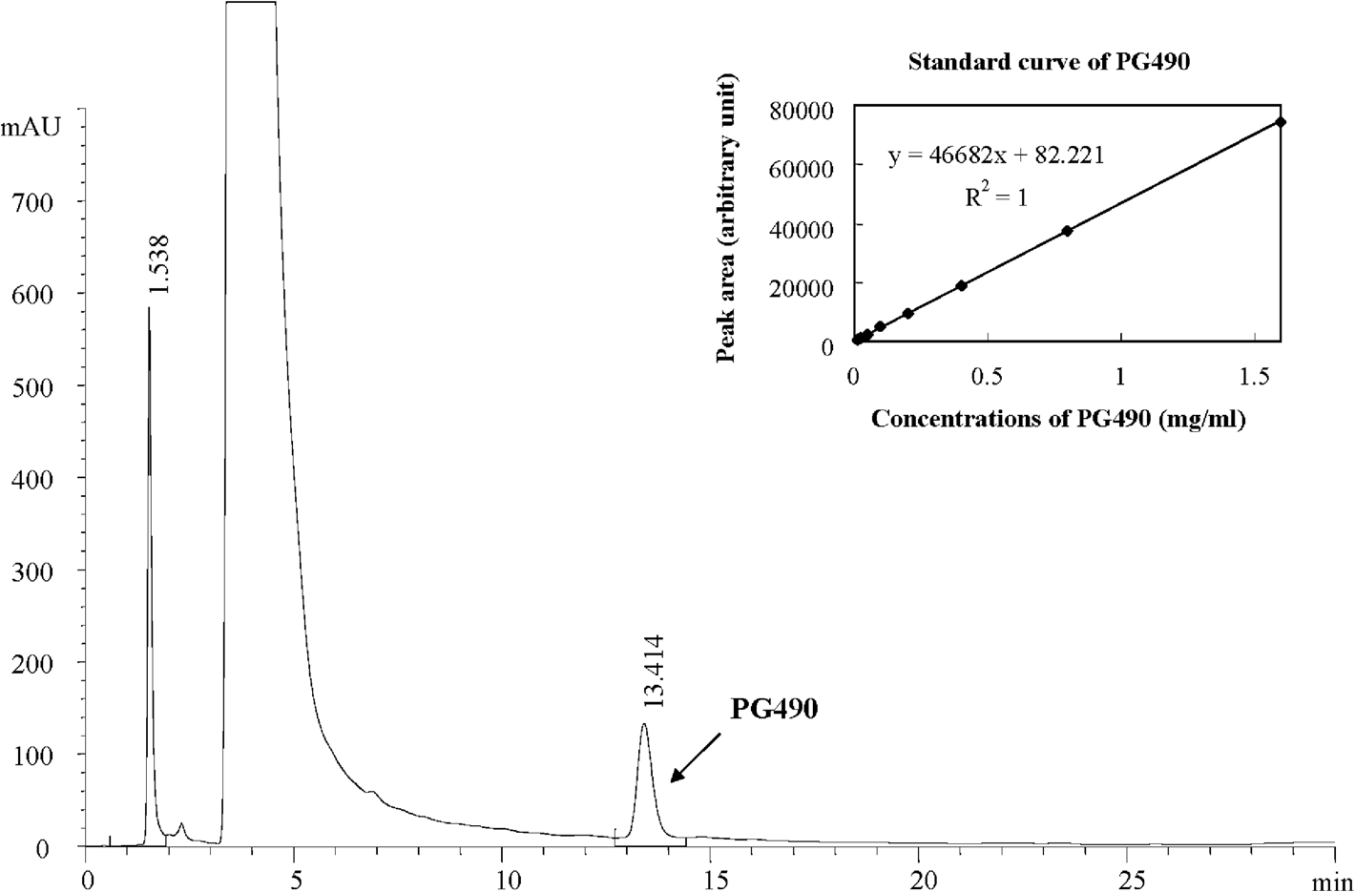
**HPLC analysis of the quantity of PG490 in PG27.** The chromatogram of PG27 with PG490 is presented, showing the retention time of 13.4 min. PG490 calibration graphs were constructed in the range of 12.5 μg/mL to 1.6 mg/mL. The regression equations of these curves and their correlation coefficients were calculated to be y = 46682x + 82.221 (r^2^ = 1, top-right corner). The peak area ratios show a linear relationship with the concentration of PG490. When the sample solution was analyzed by HPLC, the PG490 peak was confirmed by comparison to the retention time standards. The PG490 content in PG27 was determined to be 1.27 ± 0.06%.

**Figure 8:**
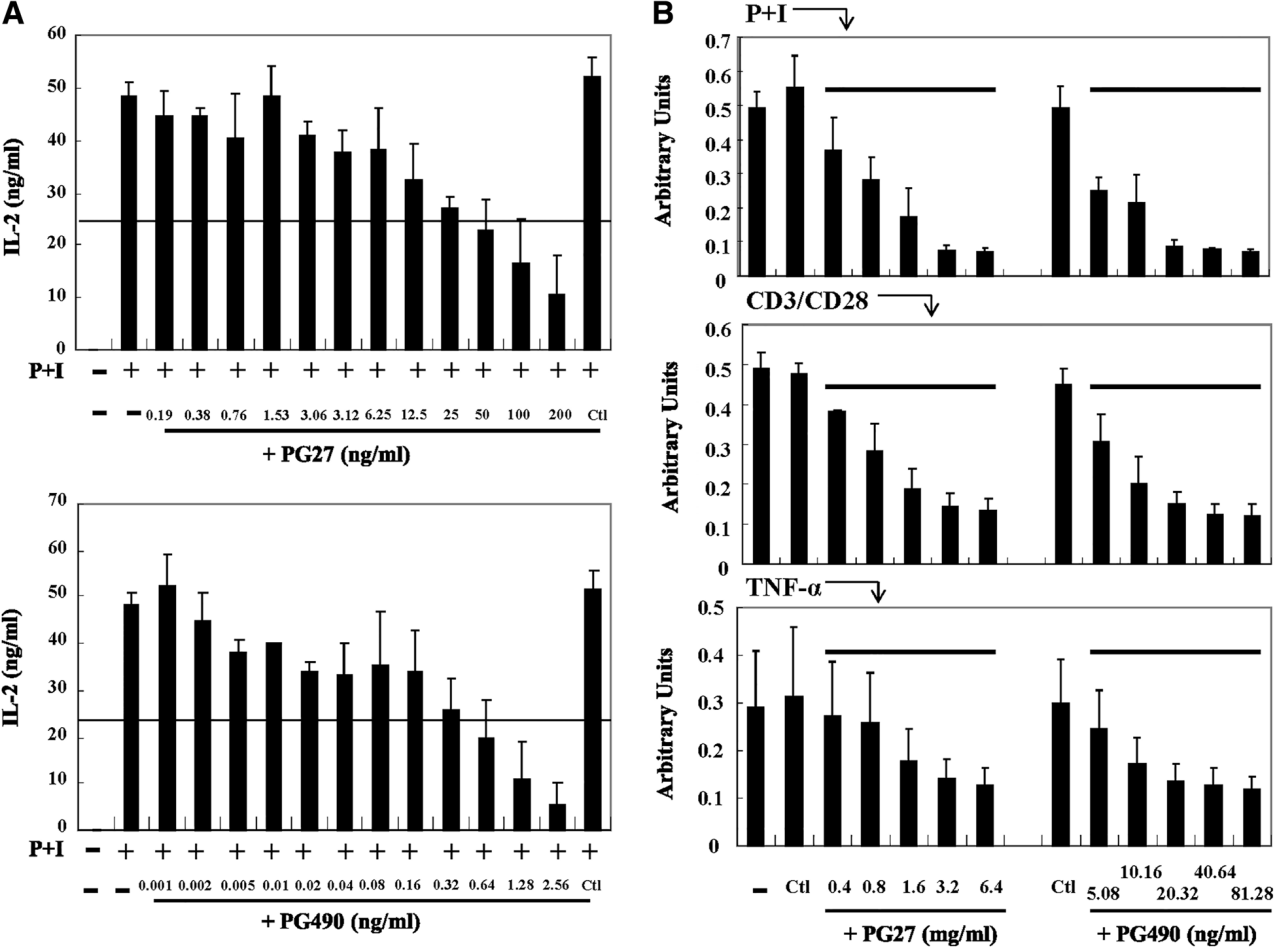
**Side-by-side comparisons of PG27 and PG490 for immunosuppressive potency and drug cytotoxicity.** T cells from a single donor were pretreated in parallel with DMSO (Ctl) or various concentrations (in serial dilutions) of PG490 or PG27 that contained an equal amount of PG490, and then stimulated with PMA + ionomycin **(A)** or 3 different stimuli (PMA + ionomycin, CD3/CD28 and TNF-α) as indicated **(B)** for 24 h. In **(A)**, the concentrations of PG27 and PG490 at 1× (the highest concentration) were 200 ng/mL and 2.56 ng/mL, respectively. In **(B)**, the concentrations of PG27 and PG490 at 1× (the highest concentration) were 6400 ng/mL and 81.28 ng/mL, respectively. The supernatants were collected for the measurement of IL-2 **(A)** and the cells were collected for the measurement of cytotoxicity by MTT colorimetric assays **(B)**. The results are representative of T cells from at least 3 different donors.

Since IKKβ activity appeared to have differential susceptibility to inhibition by PG27 and PG490, we took further steps to determine whether PG490 was a component of PG27, and if so, how much of PG27 content was constituted by PG490. We applied HPLC to examine the contents of PG27 and to determine the percentage of PG490 in PG27. As shown in Figure 7, PG490 was estimated to constitute 1.27 ± 0.06% of PG27, indicating that the highest concentration of PG27 used in this study (200 ng/mL) contained approximately 2.5 ng/mL of PG490. This quantity of PG490 was likely responsible in part for the PG27-mediated inhibitory effects on the IKK-NF-κB and MAPK-AP-1 signaling pathways. We subsequently examined whether the presence of other non-PG490 components in PG27 may affect the immunosuppressive potency and cytotoxic effects of PG490. T cells were pretreated in parallel with serial dilutions of PG490 or PG27 that contained an equivalent amount of PG490, and then stimulated with PMA + ionomycin. The concentrations of IL-2 in the culture supernatants were measured. The results revealed that the 50% inhibitory concentrations (IC_50_) for PG27 and PG490 on PMA + ionomycin-induced IL-2 production were 39.18 ng/mL and 0.19 ng/mL, respectively (Figure 8A). The IC_50_ concentrations for PG27 and PG490 on CD3/CD28-induced IL-2 production were 39.67 ng/mL and 0.23 ng/mL, respectively (Additional file 1: Figure S1). These data suggest that the low level of IKKβ-suppressive activity in PG27 did result in a partial reduction of its immunosuppressive potency, since PG490, which was shown to suppress both IKKα and IKKβ, had a greater inhibitory effect on IL-2 production. We then determined whether the cytotoxic effects of PG490 might be augmented or alleviated by other non-PG490 components of PG27. T cells were pretreated in parallel with DMSO (the solvent), PG490 or PG27 that contained an equivalent amount of PG490, and then stimulated with PMA + ionomycin, CD3/CD28 or TNF-α. The cell survival rate was determined by MTT colorimetric assays. As shown in Figure 8B, the 50% lethal concentration (LC_50_) of PG27 was higher than that of PG490 alone, indicating that the non-PG490 components of PG27 might have provided partial protection against PG490-induced cytotoxicity. The LC_50_ concentrations for PG27 and PG490 in PMA + ionomycin-stimulated T cells were 689.83 ng/mL and 4.49 ng/mL, respectively; in CD3/CD28-stimulated T cells, they were 943.8 ng/mL and 4.91 ng/mL, respectively; and in TNF-α-stimulated T cells, they were 1913.1 ng/mL and 13.9 ng/mL, respectively (Figure 8B). The higher LC_50_ concentrations in TNF-α-stimulated T cells might be due to the potential cytotoxic effects of TNF-α, which did not occur with PMA + ionomycin and CD3/CD28-stimulated T cells.

## Discussion

PG27 attracts the attention of scientists since one decade ago with its promising immunomodulatory effects in inducing antigen-specific tolerance in bone marrow transplanted mice [12] and the extending survival of cardiac xenograft models [13]. The following work was postponed because of consideration of the priority of clinical trials by the pharmaceutical company. In considering the potential applications of PG27 for autoimmune disorders, in the present study, we conducted molecular experiments to examine the mechanisms of PG27-mediated immunomodulation in human peripheral blood T cells. PG27, at therapeutic concentrations, not only suppressed various stimuli-induced transcription factor DNA-binding activities but also suppressed the transcriptional activities of both NF-κB and AP-1 (Figure 2). To our surprise, PG27 differentially regulated IKKα and IKKβ kinase activities induced by various stimuli. In contrast, the purified TwHf component PG490 inhibited both IKKα and IKKβ activities with similar potency. HPLC analysis determined that PG490 constituted 1.27 ± 0.06% of PG27 content (Figure 7). Compared to PG490 alone, PG27 that contained an equal amount of PG490 was not only less potent in immunosuppressive activity but also less cytotoxic in activated T cells (Figure 8).

Based upon the “Junn-Chenn-Zuou-SS” theory, the evidence for PG490 working as “Junn”, the main active ingredient, can be supported by its apoptosis-inducing effects and potent immunosuppressive effects demonstrated in a variety of tissue cells stimulated by different stimuli as well as in animal models of autoimmune disorders [11,14,22,23]. In addition to PG490, there are many uncharacterized components in PG27 and compared to PG490 alone, PG27 was less toxic. It seems probable that the IKKβ-suppressive effect of PG490 was masked or neutralized by other non-PG490 components in PG27, resulting in the reduction of both immunosuppressive potency and cytotoxic effects. Accordingly, some of these non-PG490 components may function as “Zuou”. Because there have been no reports simultaneously examining the combinatorial effects of two or more than two different components of TwHf, the components in PG27 that work as “Zuou” are currently unclear. Further purification and examination of PG27 components can help solve the question.

Because NF-κB transcription factors can up-regulate many genes involved in inflammatory responses, targeting NF-κB signaling events has been one of the major therapeutic goals in preventing graft rejections and in controlling autoimmune diseases [24,25]. The commonly prescribed disease modifying antirheumatic drugs also preserve inhibitory effects against NF-κB activation [26,27]. Regarding the significance of NF-κB in transplantation immunology, the inhibition of NF-κB by IκBα gene transfer is shown to improve oxygenation of the transplanted lung [28]. Transfection with NF-κB decoy into the donor lung effectively reduces lung injury during acute allograft rejections [29]. Like NF-κB, MAPK-AP-1 signaling pathway is also a critical and excellent target to block in developing therapy for inflammation-related disorders [30,31]. The inhibition of IKKα-IκBα-NF-κB and MAPK-AP-1 signaling pathways by PG27 and PG490 should lead them to potential candidates of promising immunomodulatory drugs for the therapy of autoimmune disorders and for the prevention of graft rejections.

Three major classes of stimuli, including PMA + ionomycin, the CD28 costimulatory molecule, and TNF-α, were used to activate T cells for the investigation of the immunomodulatory effects of PG27 and PG490 in human peripheral blood T cells. Different types of stimuli did not seem to affect the experimental outcomes, as the results were consistent and reproducible regardless of the stimuli being evaluated. PMA + ionomycin mimics a T cell receptor-mediated stimulus that bypasses the requirement for an antigen- or lectin-induced signal [32]. The pro-inflammatory cytokine, TNF-α, is considered to be an important molecule for the regulation of upstream cytokine cascades in inflammatory responses. The blockade of TNF-α-mediated events has been found to have significant therapeutic effects on active RA and seronegative spondyloarthropathies [33]. Given the significance of CD28 signaling in T cell activation, blockade of the CD28 signaling pathway is another promising therapeutic strategy for RA, even for those who are refractory to anti-TNF therapy [34]. Considering the critical roles of T cells in autoimmune disorders, we examined these crucial stimuli-activated T cells and demonstrated the broad-spectrum immunosuppressive capacities of both PG27 and PG490.

The differential inhibitory potency of PG27 against IKKα and IKKβ is interesting. Studies of many different compounds against IKK activity have indicated that for arthritis therapeutics, the suppression of either IKKα or IKKβ may be sufficient to block NF-κB activation [35]. The concentrations of IC_50_ for PG490 and PG27 with equivalent content of PG490 on PMA + ionomycin-induced and CD3/CD28-induced IL-2 production indicate that PG490 preserved more potent immunosuppressive activity than PG27. It suggests that the counteraction of PG490-mediated IKKβ suppression by other components resulted in reduction of immunosuppressive potency of PG27. Nevertheless, the selective suppression of IKKα but not IKKβ by PG27 may potentially lead to some benefits therapeutically. For example, in knockout studies, the targeted deletion of IKKβ results in early embryonic lethality due to extensive apoptosis of fetal hepatocytes [36,37]. In contrast, the deficiency of IKKα which results in abnormal development of skin and skeleton is relatively less fatal [38]. In addition, IKKβ plays a requisite role in B cell activation and maintenance [39]. Furthermore, the preservation of intact IKKβ-NF-κB signaling pathway is important for protecting T cells from TNF-α-induced apoptosis [38]. According to Egan et al. [40], in an *in vivo* system, the preservation of IKKβ-dependent NF-κB activation pathway is crucial for protection against radiation-induced apoptosis in intestinal epithelium. It is therefore possible that given an already suppressed IKKα-NF-κB signaling pathway, the preservation of IKKβ-NF-κB signaling pathway may help to reduce the potential side effects of PG27 in T cells and in other tissue cells. This suggestion was supported by a much reduced cytotoxic effect, as compared to PG490 alone, in PG27 containing equivalent amount of PG490 content.

## Conclusions

In this study, we observed that PG27 and PG490 had differential suppressive effects on IKKα and IKKβ activities induced by a variety of stimuli in T cells. The results also suggest that compared to PG490 alone, PG27 that contained an equivalent amount of PG490 caused less cell death. In light of the current therapeutic strategy for autoimmune disorders, which involves the combination of several disease-modifying antirheumatic drugs to increase efficacy and reduce adverse events [1], the exploration of the effects and mechanisms of Chinese antirheumatic drugs such as PG27 should provide additional alternatives for the therapy of autoimmune disorders like RA.

There are several limitations in this report. Firstly, it remains unclear the components responsible for counteracting PG490-mediated IKKβ-suppressive effects. Secondly, this *in vitro* study can not exactly reflect the *in vivo* situations, especially the situations in humans. Thirdly, whether the observations in T cells may happen in other tissue cells requires additional experiments to examine. Lastly, only a head-to-head comparison in clinical trials but not in this study can really tell us whether the observed benefit/risk of PG27 compared to PG490 does exist. Evidently, more studies are needed to answer these questions.

Traditional Chinese medicine prescriptions “Fufang” usually contain several herbs (each called “Danfang”). The traditional Chinese medicinal doctors will modify and adjust the ingredients and doses of each “Danfang” according to the need of individual patients. This formulation is based on the principle of “Junn-Chenn-Zuou-SS”. Therefore, the commonly accepted working concept of “Junn-Chenn-Zuou-SS” illustrates the specific coordinated effects from a combination of different Chinese herbs. In this study, we provide novel and interesting observations demonstrating that the “Junn-Chenn-Zuou-SS” theory may also work in a refined extract PG27 from a single herb TwHf. It is anticipated that with the inclusion of more molecular studies on Chinese herbs, the concept of “Junn-Chenn-Zuou-SS” will gain more scientific support.

## Competing interests

The authors declare that they have no competing interests.

## Authors’ contributions

LJH designed the study, performed most of the experiments of this study, and wrote the manuscript. JHL and LJH critically correct the manuscript, gave advice and guided experimental steps along the experimental process and take responsibility for correctness of the study results. WLC, AC and PC helped to perform part of the experiments and review the manuscript. All authors read and approved the final manuscript.

## Supplementary Material

Additional file 1: Figure S1 Side-by-side comparisons of PG27 and PG490 (triptolide) for immunosuppressive potency on CD3/CD28-stimulated T cells. Click here for file
