# Supplementary figures and images for: Differential immunomodulatory effects by Tripterygium wilfordii Hook f-derived refined extract PG27 and its purified component PG490 (triptolide) in human peripheral blood T cells: potential therapeutics for arthritis and possible mechanisms explaining in part Chinese herbal theory “Junn-Chenn-Zuou-SS”
Source: J Transl Med. 2013 Nov 21;11:294. doi: 10.1186/1479-5876-11-294 (PMC4222270; doi:10.1186/1479-5876-11-294)

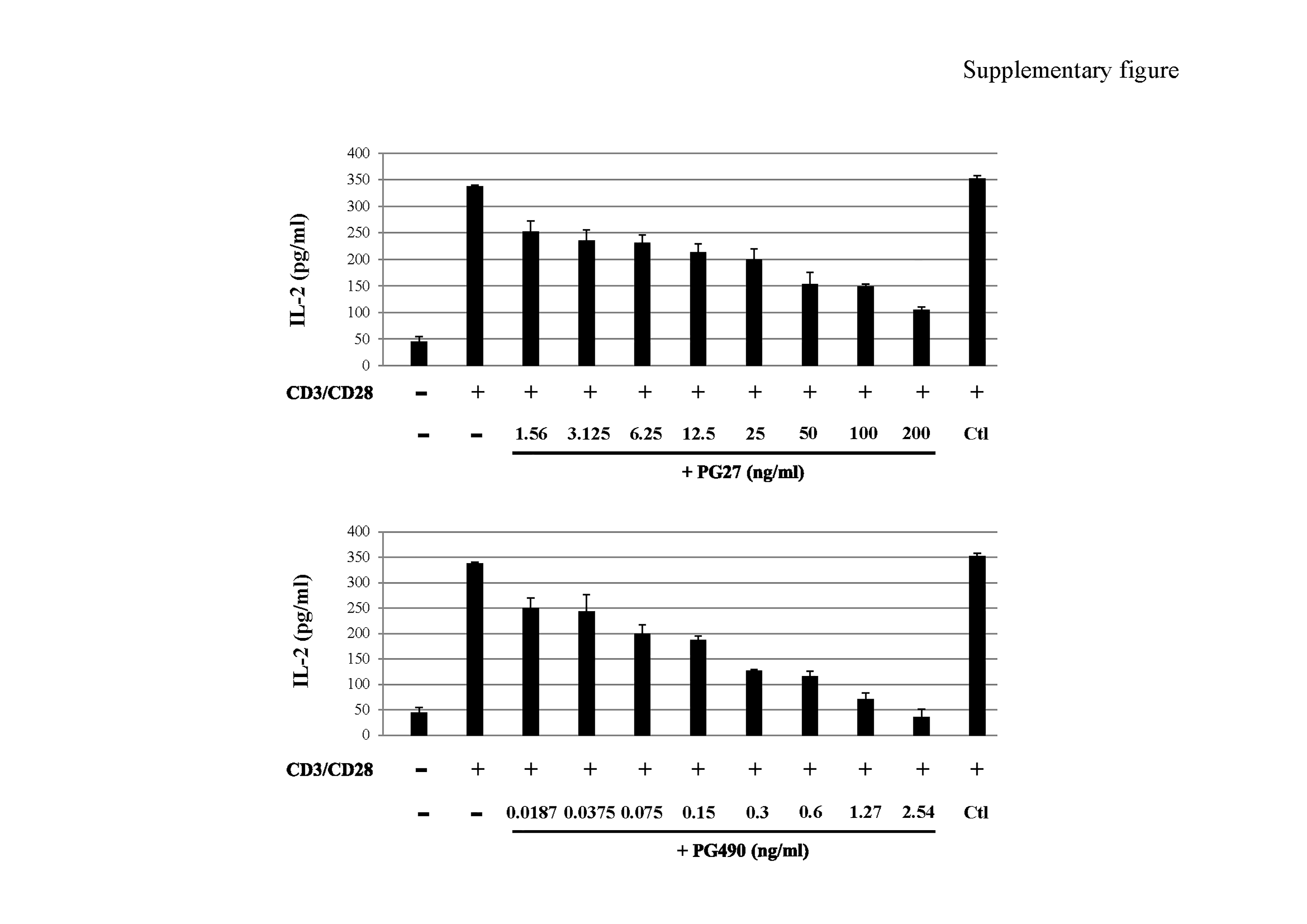

Supplement: Additional file 1: Figure S1 — Side-by-side comparisons of PG27 and PG490 (triptolide) for immunosuppressive potency on CD3/CD28-stimulated T cells. [file 1479-5876-11-294-S1.tiff]
